# Development and implementation of an electronic interface for complex clinical laboratory instruments without a vendor-provided data transfer interface

**DOI:** 10.4103/2153-3539.77176

**Published:** 2011-01-26

**Authors:** Gary E. Blank, Mohamed A. Virji

**Affiliations:** Department of Pathology, University of Pittsburgh Medical Center and the School of Medicine, Pittsburgh, PA, USA

**Keywords:** Analytical instruments, clinical laboratories, electronic interface, file transfer protocol, high-pressure liquid chromatography, laboratory information system, mass spectroscopy

## Abstract

**Background:**

Clinical pathology laboratories increasingly use complex instruments that incorporate chromatographic separation, e.g. liquid chromatography, with mass detection for rapid identification and quantification of biochemicals, biomolecules, or pharmaceuticals. Electronic data management for these instruments through interfaces with laboratory information systems (LIS) is not generally available from the instrument manufacturers or LIS vendors. Unavailability of a data management interface is a limiting factor in the use of these instruments in clinical laboratories where there is a demand for high-throughput assays with turn-around times that meet patient care needs.

**Materials and Methods:**

Professional society guidelines for design and transfer of data between instruments and LIS were used in the development and implementation of the interface. File transfer protocols and support utilities were written to facilitate transfer of information between the instruments and the LIS. An interface was created for liquid chromatography-tandem mass spectroscopy and inductively coupled plasma-mass spectroscopy instruments to manage data in the Sunquest^®^ LIS.

**Results:**

Interface validation, implementation and data transfer fidelity as well as training of technologists for use of the interface was performed by the LIS group. The technologists were familiarized with the data verification process as a part of the data management protocol. The total time for the technologists for patient/control sample data entry, assay results data transfer, and results verification was reduced from approximately 20 s per sample to <1 s per sample. Sample identification, results data entry errors, and omissions were eliminated. There was electronic record of the technologist performing the assay runs and data management.

**Conclusions:**

Development of a data management interface for complex, chromatography instruments in clinical laboratories has resulted in rapid, accurate, verifiable information transfers between instruments and LIS. This has eliminated manual data entry that is prone to errors and enabled technologists to focus on analytical applications on the instruments.

## INTRODUCTION

There has been a rapid adoption in the use of complex instruments combining liquid chromatography with mass spectrometry in hospital clinical laboratories for many applications that include identification of a variety of biological molecules, pharmaceuticals, nutraceuticals, as well as toxins. Most of these instruments do not have vendor-provided or -supported data management programs that interface with laboratory information systems (LIS). This lack of an interface makes use of the instruments cumbersome when the number and the type of analyses increase. The use of these types of instruments is projected to grow with increase in the variety of analyses that can be performed and with the transfer of selected assays from less-accurate methods to methods on these instruments that provide better precision, accuracy, and limit of detection.[1–7]

The data for analysis – sample identification, patient information, analytical components, assay results, time signatures, and technologist or operator identification – for these instruments have to be entered manually. The manual process is prone to data entry errors, utilizes operator time that could be applied to skilled functions, slows assay through-put, and increases results reporting time. These factors also limit the ability of a clinical laboratory to increase analytical capacity and assay development.

We have devised protocols and features for an interface for data management for these instruments that are based on established electronic industry and professional society standards.[8,9] The interface supports short-term data capture and audit trail of the data transfer between the analytical instruments and the LIS. The file transfer protocol (FTP) enables transfer of sample, test analysis information, and quality control data to the LIS so that the information is available directly in the LIS for further manipulation, results reporting, and archival.

## MATERIALS AND METHODS

The protocol for data handling for the liquid chromatography-tandem mass spectroscopy (LC-MS/MS) and the inductively coupled plasma-mass spectroscopy (ICP-MS) instruments for clinical laboratory use was developed based on the information that was deemed essential for positive sample identification (ID), assay results, quality control data, and the overall utility of the protocol based on the observations of the instrument operators. Assay calibration data, assay linearity verification, and mass spectral information were retained in the computer controlling each instrument and were not a part of the data transfer protocol to the LIS. The interface was designed for Sunquest^®^ LIS specifically, but could be used with modifications with other laboratory information management systems (Sunquest Information Systems Inc., Tucson, AZ, USA).

### FTP Utility

The FTP utility[10] and other support modules are written in AutoHotKey (AHK).[11] The utility consists of an executable function that manages the transfer of files to the LIS instrument manager server and an initialization (INI) file used to configure attributes of the process. The analytical instruments tested ran the Microsoft Windows^®^ XP operating system (Microsoft Corp. Redmond, WA, USA). The utility is distributed as an executable file. The program utilizes password-secured access for the compilation to ensure program integrity and to guard against reverse engineering or program modification.

Attributes configured in the INI file include the cycle time for the FTP process, all server addresses, directory and file names, data retention selections, and an optional designation of a pre-processor service program. Options allow for redirection of the files to test areas during development activities. The FTP operation by default directs all output to a “Primary” server. If a “Secondary” server is defined to serve as backup, all operations directed to the “Primary” server and any associated attributes are also assigned to the “Secondary” server.

**Figure 1:**
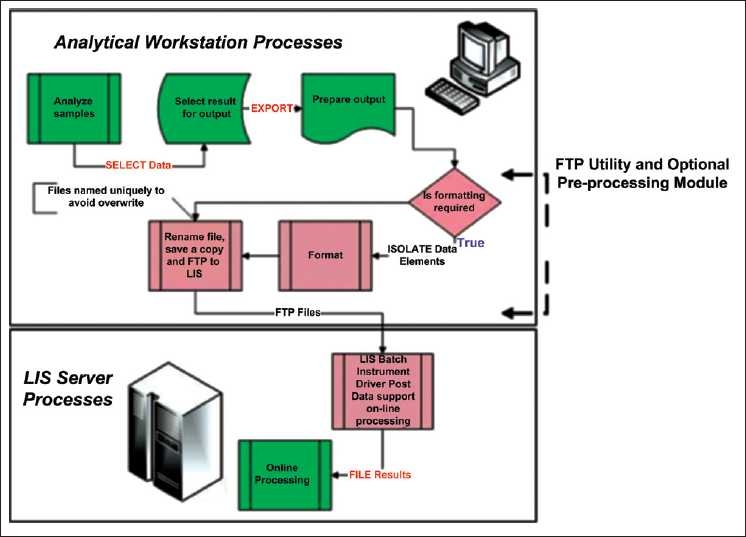
Process flow chart of local installation of the file transfer protocol utility, operator initiated, background processing

The default installation is to deploy the FTP program in a “local mode” on the personal computer (PC) controlling the analyzer [Figure 1], but can be optionally configured to run remotely from a system server. The remote configuration requires the program to be installed on a network PC mapped to a shared drive. This was not compatible with our laboratory’s requirement for activities independent of the assay performance for analytical instruments and technologists’ operations.

Once initiated via the run command, the program is self-scheduling. Many analyzers are rebooted periodically according to the vendors’ specifications. When the FTP utility is run in “local mode,” it should be added to the startup table of the analyzer’s console. An icon displaying the letters “FTP” will appear in the Windows^®^ tray and remain there as long as the program is running. A log file is maintained of the last FTP operation.

### LIS Vendor-Based Functionality

The LIS driver [Figure 1] that converts the raw data into “online” records must be configured by the LIS vendor. Additional standard parameters for an analyzer are configured through the normal LIS maintenance functions. This typically includes definitions for quality control processing and tables of analyzer identification codes to alias LIS quality control codes.

### File Pre-Processing

When the analytical instrument software is incapable of producing result files in a format compatible with the LIS vendor’s requirements, the FTP utility can schedule a “Pre-Processor” program, declared in the INI file. This is designed to convert the native instrument report file into a desired format. This can also be accomplished by designing an event-driven formatting utility operating independent of the FTP process.

### File Manipulations

The transferred files are saved for a user-defined customizable period. Older files are deleted automatically.

## RESULTS

The protocol designed was tested for accuracy of data transfer, integrity of information, completion of files transferred, and operational ease of use. All the parameters incorporated in the protocol were validated. The data transfer process is transparent to the operator as the FTP icon appears in the taskbar tray when the program is active.

1. There are three components to the data file transfer An analyzer utility on the workstation is designed to prepare a file with sample ID, test codes, and results. The data extraction modules are unique to each analyzer and are typically built around a local report generator. When the LIS is limited in acceptable flat file formats, an additional pre-processing step may be needed to achieve the desired file structure and content.
2. A utility manages the transfer by FTP of the properly formatted file to the LIS instrument manager server. This module manages all file manipulations, schedules any pre-processor, and executes the FTP command.
3. A driver on the LIS instrument manager server that parses formats and enters the data into the online processing functionality.

The three data processing steps are illustrated in Figure 1.

### 

#### Step 1; data acquisition

Convert the result data from the analyzer data reporting format into an exportable text (TXT) file with records compatible with allowed LIS Data File Structure.

If the instrument reporting software is able to create files in the desired format, no manipulations are needed and the source file can be transferred to the LIS instrument manager. Otherwise, a pre-processing step, either independently launched or started from the FTP utility, must alter the file’s data format. All file paths and names and extensions are pre-defined in the INI file.

#### Step 2; transfer the result file to LIS

Move the file by FTP transfer from the analyzer workstation source area to the LIS instrument manager server destination.

The FTP utility picks up each properly named file and renames it uniquely to avoid overwriting a file waiting the next processing step. This file is copied using the FTP protocol to the LIS server and the location appropriate for the instrument’s processing. It creates a conflict on the LIS side if the data being transmitted include prior data already sent to the instrument manager but not processed in Step 3. Because the LIS driver does not check for identical previous entries, the files successfully transferred from the analyzer are moved from the source directory to a save directory and are deleted after a customizable retention period.

A log file is maintained of the last FTP operation. The file is overwritten with each FTP attempt. The log is useful for debugging new implementations and lists server identification, file source, file sent, file destination, bytes sent, and duration of transfer.

#### Step 3; enter the data into the LIS online process

Extract the data from the flat file to the online data acquisition functionality. This process is LIS vendor specific.

The deployment of the batch style interface is designed for LC-MS/MS analyzers (Waters Inc., Milford, Mass; model 2795) and the ICP-MS (Perkin Elmer Inc., Waltham, Mass; model ELAN DRC-e). The analyses performed on the LC-MS/MS analyzers for vitamin D and for immunosuppressant (anti-rejection) drugs are both high-volume assays and include stat analyses based on clinical needs. The interfaces have been in use for 4 years with no software failures or errors since implementation.

The vendor for the LC-MS/MS analyzers provided independent software to reformat the native analyzer files to the desired layout, and no pre-processor was needed. Data conversion for the ICP-MS required a “Pre-Processor” utility for reformatting files generated by its report module. The “Pre-Processor” was written in AHK and scheduled through the FTP utility. All analyzers had the FTP utility installed locally on the instrument console and transfers were scheduled every 5 min.

During its deployment, typical technologist errors were very infrequent and limited to file name inconsistencies due to misspelling the instrument name or using the wrong instrument name designation when operating multiples analyzers in an instrument cluster performing similar analyses.

The use of the data program eliminated errors resulting from manual data management of sample ID, assay results, quality control, and assay validation. The impact of the program on the instrument operator was in reducing the data management time on the average for each sample from approximately 20 s to <1 s when running a batch of 24–28 samples. With three instruments configured for analyses of a similar group of compounds (immunosuppressant drugs), this meant that a separate individual was not required for data verification in the LIS when the information was entered manually.

## CONCLUSIONS

The data management program designed utilizing FTP has several key features that include the ability to reference a primary and a backup server, to accommodate the redirection of files to a test environment, to run unattended, to employ procedures that avoid exhausting of analyzer PC resources, and to incorporate a support module needed to reformat data to conform to LIS vendors requirements. In order to facilitate convenient changes in the associated program attributes, the values can be customized for an analytical instrument in an initialization file.

There are other features that add to the utility of the program. Setting the utility cycle time to small incremental batches, results can be available for STAT online processing in the LIS for samples designated for priority analysis. The system can be run in a “local” or remote environment, where “locally” means installation of all support modules and file storage on the analytical instrument’s computer.

In instances where the analyzer vendor objects to installation of the FTP routine on the analyzer workstation, an option is available to install the program externally. However, there are drawbacks to this form of deployment. The workstation then requires a network account. This has several implications.

- The network account must have the password updated periodically. It may be difficult to keep the technologists updated.
- Analyzers in clinical laboratories run 24 h and cannot be subject to network-imposed timeouts, such as server-scheduled reboots.
- Reboots of individual instrument consoles can produce unreliable connections.

All operations were therefore performed locally for the analytical instruments in our laboratories in order to make the entire process transparent to the instrument operators and also to avoid issues listed above.

The use of the program has many advantages. Besides eliminating key entry errors, missed data, and operator-generated sample to result mismatches, there is time saved by the operator, and this can be utilized effectively for analytical matters. Data file retention provides a useful audit trail of the technologist activities and confirmation of data synchronicity with the LIS.

The FTP process is extendable to other analyzers through an INI file customized with attributes compatible with each analyzer’s operation and the FTP utility installed on the respective system console. However, new instruments must be added to the instrument list acknowledged by the LIS instrument manager. The illustrations are for instruments utilizing mass spectrometry coupled to specific methodologies, but other areas of laboratory analyses such as molecular typing, amino acid, and DNA analysis, where standard electronic interfaces do not exist, are targets for data interface implementation using this approach.
